# Effect of Cyclic Stretch on Neuron Reorientation and Axon Outgrowth

**DOI:** 10.3389/fbioe.2020.597867

**Published:** 2020-12-14

**Authors:** Ji Lin, Xiaokeng Li, Jun Yin, Jin Qian

**Affiliations:** ^1^Key Laboratory of Soft Machines and Smart Devices of Zhejiang Province, Department of Engineering Mechanics, Zhejiang University, Hangzhou, China; ^2^State Key Laboratory of Fluid Power and Mechatronic Systems, Key Laboratory of 3D Printing Process and Equipment of Zhejiang Province, School of Mechanical Engineering, Zhejiang University, Hangzhou, China

**Keywords:** cyclic stretch, neuron reorientation, axon elongation, stress fiber, focal adhesion, microtubule

## Abstract

The directional alignment and outgrowth of neurons is a critical step of nerve regeneration and functional recovery of nerve systems, where neurons are exposed to a complex mechanical environment with subcellular structures such as stress fibers and focal adhesions acting as the key mechanical transducer. In this paper, we investigate the effects of cyclic stretch on neuron reorientation and axon outgrowth with a feasible stretching device that controls stretching amplitude and frequency. Statistical results indicate an evident frequency and amplitude dependence of neuron reorientation, that is, neurons tend to align away from stretch direction when stretching amplitude and frequency are large enough. On the other hand, axon elongation under cyclic stretch is very close to the reference case where neurons are not stretched. A mechanochemical framework is proposed by connecting the evolution of cellular configuration to the microscopic dynamics of subcellular structures, including stress fiber, focal adhesion, and microtubule, yielding theoretical predictions that are consistent with the experimental observations. The theoretical work provides an explanation of the neuron’s mechanical response to cyclic stretch, suggesting that the contraction force generated by stress fiber plays an essential role in both neuron reorientation and axon elongation. This combined experimental and theoretical study on stretch-induced neuron reorientation may have potential applications in neurodevelopment and neuron regeneration.

## Introduction

Neurons are highly specialized cells with quasi-one-dimensional processes from the cell body (named soma) on both sides that generate and transmit bioelectric impulses in nervous systems. The output end called axon is a slender neurite reaching a length over 100μ*m*, and the input end is comprised of highly branched and much shorter dendrites. It is of paramount importance in perception and motion for axons to be correctly extending to establish long-distance connections and further form a complex nervous network. Disrupted neurites in injured spinal cord and peripheral nerves can potentially lead to profound and irreversible paresis of the lower body (Silver and Miller, 2004; Case and Tessier-Lavigne, 2005; Deumens et al., 2010). There have been mounting experimental studies including mechanical (Wu et al., 2019), electromagnetic, and biochemical stimulations to improve the directional axonal outgrowth during nerve regeneration and repair (Pfister et al., 2006; Sedaghati and Seifalian, 2015).

Neurons *in vivo* are always subjected to mechanical deformation and stress in a complex manner, in association with various biological functions of their host tissue and organ. In turn, mechanical cues play an important role throughout all scales in nervous system development, from the molecular assembly in a single neuron to the final configuration of the whole system (O’Toole and Miller, 2011; Suter and Miller, 2011; Franze, 2013; Franze et al., 2013; Liu et al., 2020), indicating that neurons are sensitive to the mechanical stimulations which regulate neurite outgrowth and neurotransmitter releasing. Among them, the grooved micro-patterns employed in the substrate provide an effective approach to control the shape of neurites and the directional outgrowth of neurons (Yin et al., 2012, 2017). Recently, attention has been paid to the influence of physical forces on axon elongation that is prominent in nerve regeneration. For instance, by uniaxial drawing *in vitro*, axon tracts could be stretched to 5cm, which is a sufficient length for the core component of a nervous tissue construct (Pfister et al., 2006). O’Toole and coworkers (O’Toole et al., 2008a; O’Toole and Miller, 2011) incorporated the effects of stretch in a model of slow axonal transport, quantifying the impact of stretching on axon elongation. Ishibashi et al. (2016) studied the enhancement of cyclic stretch on dorsal root ganglion (DRG) neuron outgrowth, demonstrating that the stretching duration rather than the frequency is an influential factor. This can be understood in such a way that the influence of cyclic stretch on axon elongation corresponds to that of continuous stretch with an equivalent stretching level and time. Interestingly, Higgins and coworkers (Higgins et al., 2013) found that equi-biaxial dynamic stretch with an average value of zero (sinusoidal stretch at 10% strain and 0.25 Hz) effectively enhanced axon elongation. Furthermore, the effect of cyclic stretching on neuron/axon elongation could be amplified with the aid of electromagnetic stimulation (Nakamachi et al., 2018) or micro-textured substrates (Chang et al., 2013). Besides this, cyclic tensile stress was also reported to impact on the expression of genes associated with neuronal cell death (Uchida et al., 2008, 2010).

Compared to the elaborate works on stretching-stimulated neurite outgrowth, the alignment or orientation of neuron was less studied but was indispensable in nerve regeneration. Neurons were reported to orientate along the direction of the applied strain when they are cultured on the substrate subjected to continuous stretching (Chang et al., 2013). The neurite orientation of rat pheochromocytoma cells has been studied, with the ambiguous conclusion that cells can orientate in either parallel direction or perpendicular direction under different combinations of stretching level and frequency (Haq et al., 2006). On the other hand, intensive studies showed that different types of cells, such as fibroblasts (Eastwood et al., 1998; Jungbauer et al., 2008; Goldyn et al., 2010), osteoblasts (Neidlinger-Wilke et al., 1994; Wang et al., 2000), melanocytes (Wang et al., 1995), muscle cells (Stavenow, 1986; Collinsworth et al., 2000), and endothelial cells (Moretti et al., 2004), tend to align nearly perpendicular to the stretch direction when cultured on a substrate with oscillating uniaxial strain at relatively high frequency (>1 Hz) and stretching magnitude (>5% strain). The consistent behavior of various cell types under cyclic stretching suggests that cell reorientation may be governed by a common physical mechanism. There have been several theoretical models that work on cell reorientation (Wang, 2000; De et al., 2007, 2008; Qian et al., 2013; Livne et al., 2014; Chen et al., 2015; Xu et al., 2016). However, whether or not neurons have an analogical response to cyclic stretching remains an elusive issue.

In this paper, we perform a comprehensive study through experimental observation and theoretical modeling to investigate the role of cyclic stretch on the process of neurite elongation and neuron reorientation. We firstly design and assemble a stretching system where neurons adhere to the polydimethylsiloxane (PDMS) substrate subjected to controllable clamps. We evaluate the effect of cyclic stretching stimulation on cell reorientation and axon elongation by adopting relatively low values of stretching amplitudes (2–10%) and frequencies (0.05–0.25 Hz) that will not cause neuron death (Tanoue et al., 1996). The number of neurons and neurite length are found to increase with culture time, and the statistical results of cell alignment and axon length are extracted from experimental images. Based on the experimental results, we propose a theoretical framework that accounts for the dynamic evolution of subcellular structures such as focal adhesions (FAs) and stress fibers, explaining the cell alignment and further relating the axon elongation to the assembly of microtubules and tubulin transportation driven by intracellular traction that is generated by stress fibers. Our systematic study on the response of neurons to cyclic stretching demonstrates that cyclic stretch rearranges neurons but hardly affects axon elongation. These combined experimental and theoretical results may provide a guideline for nerve repair and neurology.

## Materials and Methods

### Stretching Device

The substrate was manufactured by PDMS that is widely used as a biomaterial due to its biocompatibility, sterilizability, as well as mechanical, thermal, and chemical properties (Moretti et al., 2004). In detail, PDMS (Sylgard 184; Macklin, China; elastomer/cross-linker = 10:1) was spun onto a glass plate to form a film with a thickness of 1 mm; then, the film was cured in an oven at 80°C for 2 h. The PDMS film was peeled off from the glass plate and cut into 20 × 60-mm strips. After sterilization, these strips were soaked in poly-L-lysine (Beyotime) for 30 min and then dried to improve the biocompatibility.

**FIGURE 1:**
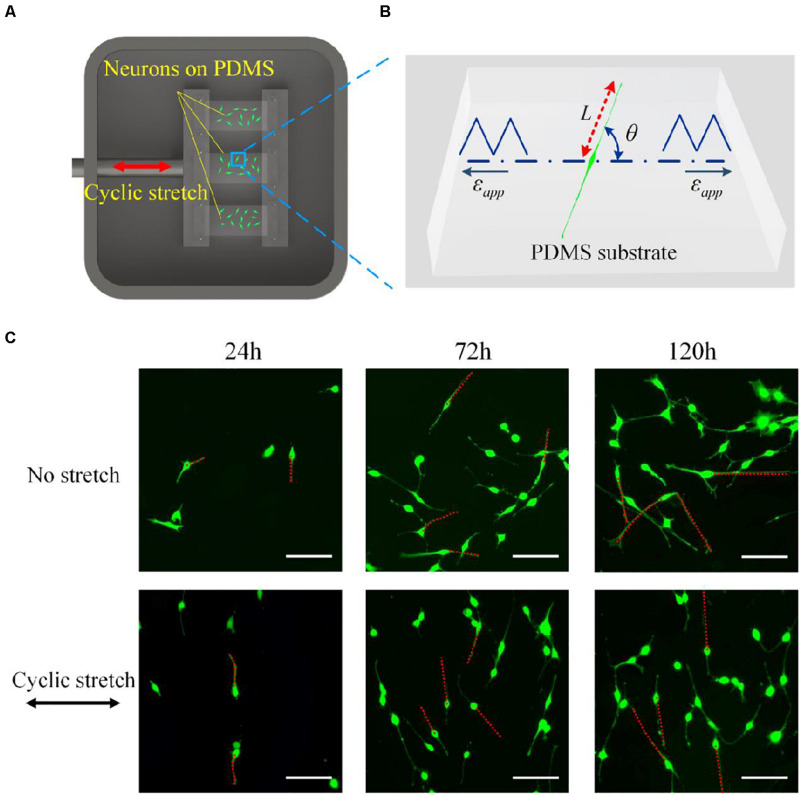
**(A)** Schematic drawing of the experimental setup. An elastic polydimethylsiloxane film is clamped and cyclically elongated in the direction of the red arrow. **(B)** Snapshot of a PC12 cell adhered to a substrate that is subjected to cyclic stretch ε_app_. *L* is the length of the axon, and θ is the orientation of the neurite outgrowth, which is defined as the angle between axon alignment and stretch direction. **(C)** Morphology images of PC12 cells after 24, 72, and 120 h under no stretch (upper row) and cyclic stretch (lower row) with 10% amplitude and 0.25 Hz frequency. Scale bar: 100 μm.

The stretching device (CellScale MechanoCulture T6) was used to stretch the PDMS substrates, which mainly contained a chamber of cultured cells, including a fixed clamp, and a mobile clamp that is connected to the motor by a stainless steel bridge. The motor was controlled by a circuit board (controller) that can be programmed to separate the clamps with a prescribed amplitude and frequency. As soon as the motor was started, the substrate was uniaxially stretched (in the direction marked with a red arrow in Figure 1A) in a pre-set mode.

PC12 cells (Cell Bank of the Chinese Academy of Sciences, Shanghai, China) with a density of 8 × 10^4^ cells per milliliter were placed onto the surface of PDMS strips for 4 h. Then, the PDMS strips were transferred into the chamber with 100 ml Dulbecco’s modified Eagle’s medium (DMEM) and connected to clamps for a cyclic stretch in the periodic form:

(1) $$\varepsilon_{\text{app}}\left(t\right)=\left\{ \begin{array}{cc} 2\varepsilon \left(\omega \text{t-}n\right), & \frac{n}{\omega }\leq t<\frac{n}{\omega }+\frac{1}{2\omega }\\ 2\varepsilon \left(n+1-\omega t\right), & \frac{n}{\omega }+\frac{1}{2\omega }\leq t\leq \frac{n+1}{\omega } \end{array}\right.,$$

where ε is the stretching amplitude determining the maximum magnitude of stretch (Figure 1B), ω is the frequency, and *n* denotes a natural number. In Table 1, we show different combinations of stretching frequency and amplitude in the experiments, with the frequency ranging from 0.05 to 0.25 Hz and amplitude from 2 to 10%. The clamped substrate was slightly pre-stretched to avoid possible wrinkles on the substrate surface.

**TABLE 1:** Values of stretching frequency and amplitude used in the experiments.

	1	2	3	4	5	6
Frequency (Hz)	No stretch	0.05	0.15	0.25	0.25	0.25
Amplitude (%)		10	10	10	2	5

### PC12 Cell Culture

PC12 cell has been widely used as a suitable model for investigating the biomedical and functional properties of neuronal cells *in vitro* (Suo et al., 2018) due to the similarity of well-differentiated PC12 cells to nerve cells in terms of morphology, physiology, and biochemistry. The PC12 cells were cultured in DMEM (Hyclone, China) supplemented with 10% fetal bovine serum (Gibco, United States) in an incubator (Thermo Scientific, United States) at 37°C with 5% CO_2_. The PC12 cells, with a concentration of 8 × 10^4^ cells/ml, were harvested from cell culture dishes and transferred onto the PDMS substrates for 4 h. Then, the PDMS substrates were transferred to the stretching device and stretched for 120 h.

### Cell Stain and Morphology Observation

The cultured PC12 cells were stained by Calcein-AM (Aladdin, China) for morphology observation, where the PDMS substrates were rinsed with PBS (Aladdin, China) and incubated with 1 μg/ml Calcein-AM for 30 min to stain PC12 cells after stretching for 24, 72, and 120 h, respectively. The cells on PDMS substrates were observed and imaged using a fluorescence microscope (Ti-S, Nikon, Japan). The morphology of neurons was quantified in terms of the length (*L*) and the overall orientation (θ) of neurite outgrowth, as shown in Figure 1B. *L* is defined as the end-to-end distance from soma to axon tip, and θ is defined as the angle between the axon and the stretch direction.

In the statistical analysis, the distribution of orientation θ was further divided into 12 uniform intervals with a span of 30°. Statistical analysis was implemented using unpaired Student’s *t*-test, and *P*-value < 0.05 (^∗^) was adopted as the significant difference. Each data of axon length and cell orientation was obtained based on the measurements of more than 100 PC12 cells *via* the image processing software ImageJ (National Institutes of Health, United States).

## Experimental Results

To investigate the influence of cyclic stretch on cell response, we adopted amplitude ε = 10% and frequency ω = 0.25Hz in Eq. (1), and cells are also cultured on the substrate without stretch for comparison (no-stretch group). Two rows of images in Figure 1C show snapshots of PC12 cells at different times under no stretch and cyclic stretch, respectively. We can see that PC12 cells orient randomly in the no-stretch group and gradually reorient to the perpendicular direction of stretch under cyclic stretch. These findings agree with many previous reports (Stavenow, 1986; Neidlinger-Wilke et al., 1994; Wang et al., 1995; Eastwood et al., 1998; Collinsworth et al., 2000; Wang et al., 2000; Moretti et al., 2004; Jungbauer et al., 2008; Goldyn et al., 2010) that various cell types tend to reorient themselves away from the stretch direction. At the same time, we find that the neurite outgrowth (axon length) of neurons increases with time, regardless of cyclic stretch.

### Cell Reorientation Depending on Cyclic Stretch

**FIGURE 2:**
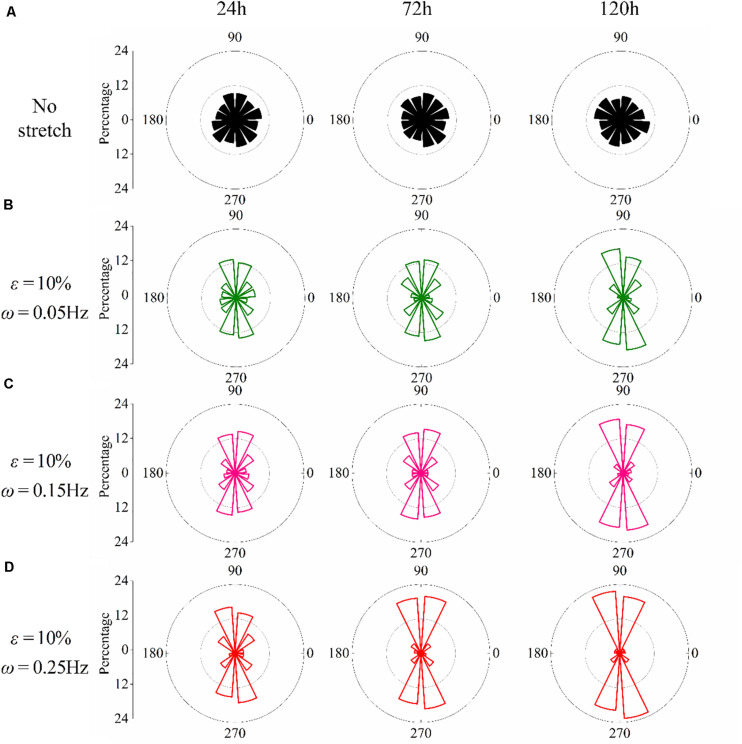
Statistical results of PC12 cell reorientation at different frequencies after 24, 72, and 120 h. **(A)** No stretch, **(B)** ω = 0.05Hz, **(C)** ω = 0.15Hz, and **(D)** ω = 0.25Hz. The stretching amplitude is fixed at 10%.

We have further studied the frequency dependence of PC12 cells by cyclically stretching the substrate with three different frequencies: ω = 0.05Hz, ω = 0.15Hz, and ω = 0.25Hz. Corresponding to the snapshots in Figure 1C, we collect the orientation of each neuron for the no-stretch case in Figure 2A, finding that the percentage of cells located in each interval is around 9% throughout the whole process (24, 72, and 120 h). In contrast, with cyclic stretch, cells dramatically polarize, and most of the cells are oriented in the ranges of 60≤∘θ≤120∘ and 240≤∘θ≤300∘ (Figures 2B–D). The percentage of perpendicularly polarized cells increases with elapsed time, especially when ω = 0.25Hz (Figure 2D). The percentage of cells that orientate away from the stretch direction is 62.63% at 24 h and increases to 75.58 and 83.81% at 72 and 120 h, respectively. At every moment, we can find more cells in the orientations of 60≤∘θ≤120∘ and 240≤∘θ≤300∘ when the frequency is higher, comparing the statistical results in Figures 2B–D.

**FIGURE 3:**
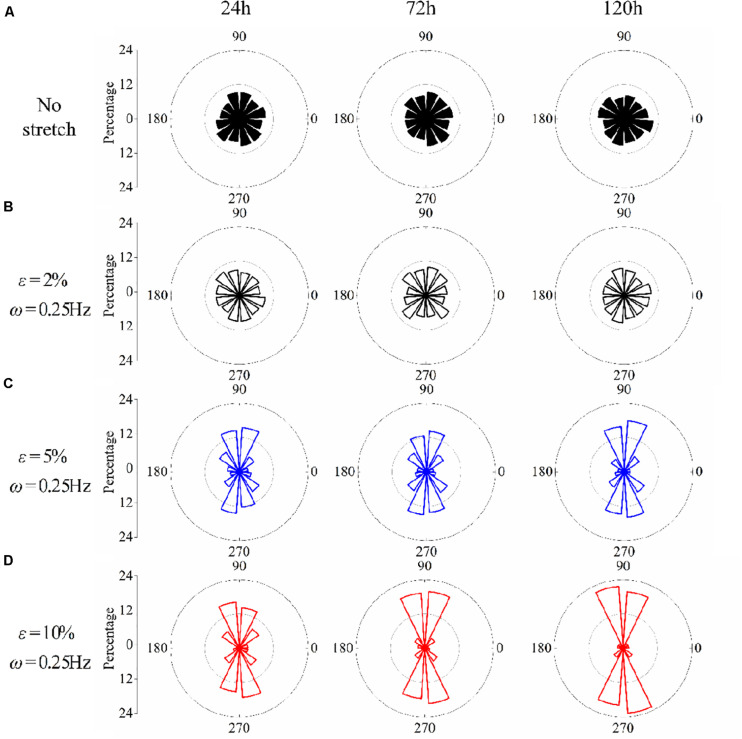
Statistical results of PC12 cell reorientation at different amplitudes after 24, 72, and 120 h. **(A)** No stretch, **(B)** ε = 2%, **(C)** ε = 5%, and **(D)** ε = 10%. The frequency is fixed at ω = 0.25Hz.

The effect of stretch amplitude on neuron reorientation is also examined by choosing three different amplitudes of ε = 2%, ε = 5%, and ε = 10%, while the frequency is fixed at ω = 0.25Hz. For the two cases of zero stretch (Figure 3A) and ε = 2% (Figure 3B), the cells are nearly uniformly distributed in all the orientation intervals. When the amplitude exceeds 5% (Figure 3C), the neurons prefer to stay in the perpendicular direction of the applied stretch, and this tendency is more remarkable when ε = 10% (Figure 3D). The exact percentages of cell in the angle intervals (60≤∘θ≤120∘ and 240≤∘θ≤300∘) of 10% amplitude and 5% amplitude are 62.63 and 52.51% after 24 h, 75.58 and 53.61% after 72 h, and 83.81 and 64.21% after 120 h, respectively. The results also indicate that PC12 cells reorient to the perpendicular direction of stretch faster with a higher polarization level at a higher stretch amplitude (when stretch amplitude is higher than 5%).

### Neurite Outgrowth Insensitive to Cyclic Stretch

**FIGURE 4:**
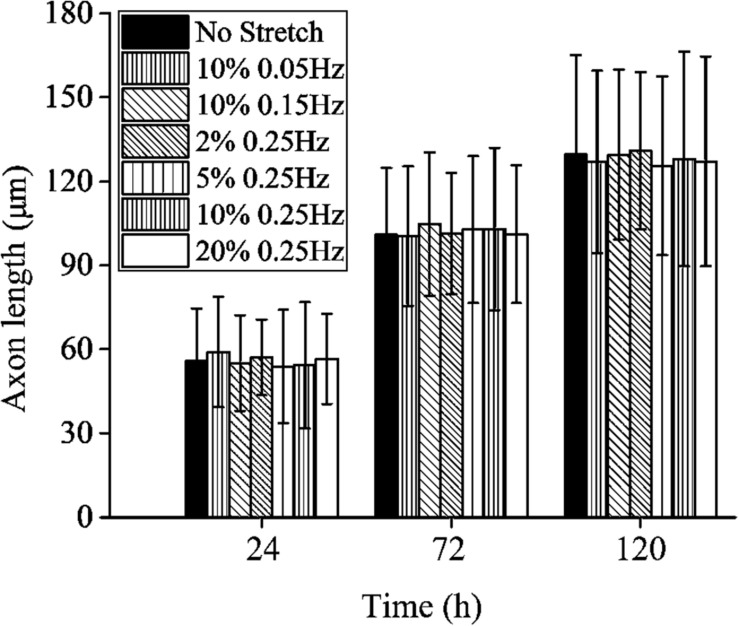
Axon length of PC12 cells with no stretch and cyclic stretch after 24, 72, and 120 h. Error bar stands for standard deviation (*n* = 100).

Contrary to the stretch-induced reorientation of PC12 cells, the imposed stretch does not influence the outgrowth of neurite of PC12 cells. We summarize the mean axon length of PC12 cells at time of 24, 72, and 120 h (Figure 4), where all the different stretch conditions nearly lead to the same axon elongation. The variation of the axon length is always below 5% when the frequency of cyclic stretch changes from 0.05 to 0.25 Hz, and the amplitude changes from 2 to 20%. The observation here is different from that of the previous studies showing that the dynamic stretch can effectively enhance axon elongation (Higgins et al., 2013; Ishibashi et al., 2016). The difference is originated from the mean value of imposed cyclic stretch: in the present work, the applied stretch is a wavy function with an average strain of zero, while in the previous studies (Higgins et al., 2013; Ishibashi et al., 2016), the applied strain varies from 0 to 10%, with an average value of 5%. Therefore, our study indicates that cyclic stretch with zero mean has negligible effects on axon elongation.

## Model Formulation

### Structural Modeling of Stress Fiber

To capture the experimental scenario where a neuron cell adheres to an elastomeric substrate subjected to a cyclic tensile strain ε_*app*_ (Figure 1A), we first approximate the imposed cyclic strain with a sinusoidal form, namely,

(2) $$\varepsilon_{\text{app}}\left(t\right)=\frac{\varepsilon }{2}\left(1+\text{sin}\left(\omega t+\varphi \right)\right).$$

**FIGURE 5:**
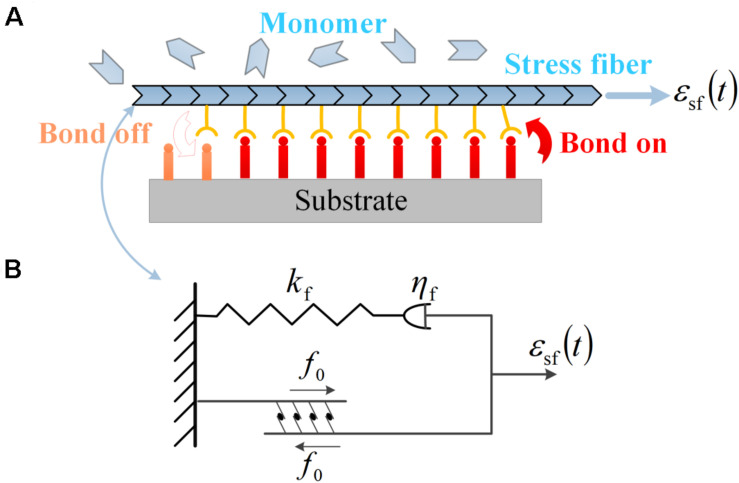
**(A)** Subcellular structures of neurons under cyclic stretch: a stress fiber in PC12 cell adhered to the substrate *via* a focal adhesion of receptor–ligand bonds. The evolution of the bond cluster is governed by the association and dissociation of receptor–ligand bonds. The stress fiber is assembled by monomers and can retrograde into monomers. **(B)** The viscoelastic model of contracting stress fiber described by a linear spring of stiffness *k*_f_, a dashpot of viscous coefficient η_f_ in series, and a parallel module of active contraction force *f*_0_.

Same as those in Eq. (1), ε represents the amplitude of stretch, and ω stands for the frequency. $\varphi =-\frac{\pi }{2}$ is a phase angle that ensures the initial condition that zero strain is applied to substrate at *t* = 0. Neuron cells arrayed on the surface of the substrate are mediated by FAs, which connect bundles of actin filaments (also called stress fibers) to the extracellular matrix along the alignment of the axon (Liu et al., 2020). Through FAs, the traction and deformation acting on the substrate are transformed to the neuron cells, as described in Figure 5A. According to the transformation law of deformation tensor, when the neuron cell is oriented at an angle θ with respect to the stretch direction, the effective stretching strain acting on the stress fiber is related to the applied strain to the substrate through

(3) $$\varepsilon_{\text{sf}}=\varepsilon_{\text{app}}{\text{cos}}^{2}\theta .$$

It should be noted that the orientation angle of the axon (θ, the angle between the axon and the stretch direction) is quantified for the alignment behavior of neurons, consistent with the preceding experiments; the soma part of neurons is assumed invariant in the present modeling, and it is the axon part (combining stress fiber and microtubule) that determines the reorientation dynamics of neurons in the following; the growth cone is assumed to have the same alignment as the axon under cyclic stretch.

The mechanical responses of SF to external stretch can be commonly represented by a Maxwell viscoelastic model (Deguchi et al., 2006; Kumar et al., 2006), where an elastic spring with stiffness *k*_f_ is linked to a viscous damper with viscous coefficient η_f_ in series. Under this circumstance, the strain ε_sf_ applied on the stress fiber enters into the spring and dashpot, denoted as ε_1_(*t*) and ε_2_(*t*), with a conservation law of strain and force balance between the two parts as the following:

(4) $$\varepsilon_{1}\left(t\right)+\varepsilon_{2}\left(t\right)=\frac{\varepsilon }{2}\left(1+\text{sin}\left(\omega t+\varphi \right)\right){\text{cos}}^{2}\theta ,$$

(5) $$k_{\text{f}}\varepsilon_{1}\left(t\right)=\eta_{\text{f}}\frac{\text{d}\varepsilon_{2}\left(t\right)}{\text{d}t}.$$

Solving these coupled governing equations [Eqs (2–5)], we obtain the passive force generated in the stress fiber in response to the imposed stretch:

(6) $$\begin{array}{c} f_{\text{s}}=k_{\text{f}}\varepsilon_{1}\left(t\right)=\frac{k_{\text{f}}\varepsilon }{2}(\left(1-\frac{\alpha }{1+\alpha^{2}}\right)e^{-\alpha \left(\omega t+\varphi \right)}\\ \;+\frac{\alpha }{1+\alpha^{2}}\text{cos}\left(\omega t+\varphi \right)+\frac{1}{1+\alpha^{2}}\text{sin}\left(\omega t+\varphi \right)), \end{array}$$

where α = *k*_f_/(ωη_f_) is a dimensionless ratio. In addition, due to the activity of associated motor proteins, SF can generate active force which can be regarded as a constant value of *f*_0_. Therefore, the total force in the stress fiber is the summation of *f*_0_ and *f*_s_, as depicted in Figure 5B:

(7) $$f=f_{0}+f_{\text{s}}.$$

This description immediately reduces to the well-known Hill’s model for the filament contraction in muscles (Hill, 1938; Wei et al., 2008) if we neglect the elastic spring in the model.

### Kinetics of FA and SF Assemblies

The waveform force generated within the stress fiber [Eqs (6, 7)] is present throughout the dynamics of neuron cells and governs the formation or disassembly of subcellular structures. We describe the evolution of focal adhesion and stress fiber by monitoring the time-varying behaviors of bond density ρ_*b*_ in the focal adhesion, as well as the density of contracting filament ρ_*f*_ in the stress fiber, which obey the first-order kinetic equations (Qian et al., 2013) as

(8) $$\frac{\text{d}\rho_{\text{b}}}{\text{d}t}=k_{\text{on}}\left(\rho_{0}-\rho_{\text{b}}\right)-k_{\text{off}}\rho_{\text{b}},$$

(9) $$\frac{\text{d}\rho_{\text{f}}}{\text{d}t}=k_{\text{on}}^{\text{f}}\rho_{\text{b}}-k_{\text{off}}^{\text{f}}\rho_{\text{f}}.$$

Here ρ_0_ is the theoretical maximum value of bond density that can be achieved in FA. *k*_on_ and *k*_off_ stand for the association and dissociation rates of molecular bonds in FA, while $k_{\text{on}}^{\text{f}}$ and $k_{\text{off}}^{\text{f}}$ represent the on- and off-rate of contracting filaments in SF, respectively. Note that the forward rate of filament growth is assumed to be proportional to the receptor–ligand bond density ρ_*b*_ [referring to Eq. (9)], which is consistent with the observed correlation between the formation of FAs and SFs in experiments (Balaban et al., 2001; Novak et al., 2004).

The rates *k*_on_, $k_{\text{on}}^{\text{f}}$, and $k_{\text{off}}^{\text{f}}$ are regarded as constants throughout this work, and the bond dissociation rate *k*_off_ is assumed to be related to the energy reduction, *G*, in forming a single receptor–ligand bond through the form:

(10) $$k_{\text{off}}=k_{\text{off}\_0}\text{exp}\left(-\frac{G}{k_{\text{B}}T}\right),$$

with *k*_off_0_ being the dissociation rate of receptor–ligand bonds under membrane fluctuations and steric repulsion of glycocalyx, in the absence of SF. Considering the fact that no stable focal adhesion can be formed in the absence of SF, *k*_off_0_ should adopt a much larger value than *k*_on_. The energy reduction *G* is scaled by the unit of thermal energy *k*_B_*T*, with *k*_B_ being the Boltzmann constant and *T* being the ambient temperature in degrees Kelvin, and we adopt a plausible expression of such energy reduction as

(11) $$G=ak_{\text{B}}T\frac{\rho_{\text{f}}}{\rho_{\text{b}}}-\frac{f^{2}}{2K}{\left(\frac{\rho_{\text{f}}}{\rho_{\text{b}}}\right)}^{2},$$

where the first term on the right-hand side corresponds to the interaction energy between the receptor–ligand bonds and the reinforcing proteins in FA/SF complex, with a positive coefficient *a* representing single-pair interaction energy in units of *k*_B_*T* and an efficient ratio ρ_f_/ρ_b_ representing the interaction between receptor–ligand bonds and contracting filaments. The second term accounts for the elastic energy stored in the system of receptor–ligand bonds and extracellular matrix, in the presence of contracting force from SF. *f* is defined as the contracting force generated by each filament in SF, as shown in Eq. (7). Therefore, *f*⋅ρ_f_/ρ_b_ stands for the average load supported by individual receptor–ligand bonds. *K* is an effective spring constant representing the combined stiffness of a bond–substrate system and is related to the combined effective modulus of the substrate and the bond in the form of *K* = *E*δ, with δ being a length scale characterizing the lateral size of a receptor–ligand bond (with a typical value of 10 nm; Hynes, 1992; Arnold et al., 2004).

### Dynamics of Cell Reorientation and Involved Time Scales

To maintain a stable configuration, cells are required to develop long-term stability in both focal adhesion and stress fiber. The maintenance and development of focal adhesion have been found to be profoundly influenced by the elastic modulus (Qian et al., 2008, 2009), anisotropy (Zhang et al., 2013), morphology (Yu et al., 2017), and chirality (Dong et al., 2019) of the substrate. Under cyclic stretch, the force generated within cells can increase to beyond a threshold value, giving rise to elastic energy in the bond–substrate system, thereby increasing the dissociation rate of receptor–ligand bonds. In this way, neurons in some orientations may fail to develop stable SFs and FAs and must undergo rotational diffusion at random to explore other possible orientations for nucleation and formation of new FAs and polymerization of new stress fibers. In this study, the dynamics of cell reorientation is thus described as a loop of orientation search, focal complex nucleation, and FA/SF development, which repeats until a neuron finds a certain alignment, allowing itself to maintain stable FA and SF structures. The random rotational diffusion of the whole neuron can be described by the classical model of Brownian motion, i.e., dθ^2^ = 2*D*_r_*t*^∗^. Here θ is the orientation angle, *D*_r_ is the rotational diffusion coefficient in units of rad^2^/s, and *t*^∗^ is the time interval between losing adhesion at old orientation and nucleating adhesion at new orientation. In an alternative description of equivalence, a neuron cell will hop by an angle $\text{d}\theta =N\left(0,1\right)\sqrt{2D_{r}t^{*}}$ in each orientation search, with *N*(0, 1) being a random number following normal distribution with zero mean and unit variance.

### Elongation Process of Neuron Axon

Neurons germinate the axon during their reorientation process under cyclic stretch *via* the assembly of microtubules, which can be modeled by coupling the transportation of monomers (known as tubulin dimers) and assembly/disassembly kinetics of microtubules. The tubulins are generated in the cell body (also named soma) and subsequently conveyed to the tip of the axon (named growth cone), where microtubule elongation resulting from tubulin assembly facilitates the progress of the growth cone. To simplify the model that captures the primary biophysical features of axon elongation, the elongation of the axon is assumed to be the same as the increase of the microtubule length, which is governed by the assembly/disassembly of microtubules in the growth cone (Diehl et al., 2014; Purohit and Smith, 2016):

(12) $$\frac{\text{d}\left(A_{\text{MT}}l\right)}{\text{d}t}=\tilde{r_{\text{g}}}V_{\text{c}}c_{\text{c}}-\tilde{s_{\text{g}}}A_{\text{MT}}l_{0},$$

where *A*_MT_*l* is the volume of microtubules, with *A*_*MT*_ being the effective cross-sectional area and *l* being the length of the microtubules. The first term on the right-hand side corresponds to the assembly of microtubules in the growth cone, which is proportional to the amount of free tubulin *V*_c_*c*_c_ (*V*_c_ is the volume of the growth cone, and *c*_c_ is the concentration of tubulin) in the growth cone, with a rate of $\tilde{r_{\text{g}}}$. The second term stands for the disassembly with reducing rate, $\tilde{s_{\text{g}}}$, being proportional to the assembled microtubules *A*_MT_*l*_0_, where *l*_0_ is the length of the assembled microtubules in the growth cone. Assuming that the cross-sectional area of microtubules (*A*_MT_) is a constant, we can obtain the governing equation of axon length: $\text{d}l/\text{d}t=\tilde{r_{\text{g}}}Vc_{\text{c}}/A_{\text{MT}}-\tilde{s_{\text{g}}}l_{0}$, which can be rewritten as $\dot{l}\left(t\right)=r_{\text{g}}c_{\text{c}}-s_{\text{g}}$. The denotation $r_{\text{g}}=\tilde{r_{\text{g}}}Vc_{\text{c}}/A_{\text{MT}}$ can be regarded as an effective rate coefficient for polymerization and $s_{\text{g}}=\tilde{s_{\text{g}}}l_{0}$ can be interpreted as the maximum speed of shrinkage, and it is reasonable to define *r*_g_ and *s*_g_ as constant parameters for exploring the essential dynamics. The axon stops elongating when the concentration of the tubulins is below a threshold value *c*_∞_ = *s*_g_/*r*_g_, so we get the rate of axon elongation in the following form:

(13) $$\frac{\text{d}l}{\text{d}t}=r_{\text{g}}\left(c_{\text{c}}-c_{\infty }\right).$$

The concentration of free tubulins in the growth cone (*c*_c_) is affected by the assembly and disassembly of microtubules, degradation of tubulins, and compensation of the newly generated tubulins in soma. The conservation of the amount of free tubulins requires that

(14) $$\frac{\text{d}\left(V_{\text{c}}c_{\text{c}}\right)}{\text{d}t}=AJ_{\text{c}}-A\frac{\text{d}l}{\text{d}t}c_{\text{c}}-gV_{\text{c}}c_{\text{c}}-\tilde{r_{\text{g}}}V_{\text{c}}c_{\text{c}}+\tilde{s_{\text{g}}}A_{\text{MT}}l_{0}.$$

Here *AJ*_c_ is the influx of tubulins that are generated in the soma, with *A* standing for the cross-sectional area of the axon and *J*_c_ being the flux term. The second term arises from the moving boundary of the growth cone, which is assumed to be identical to the elongation speed of the axon. The free tubulins also degrade at a rate of *g*, decreasing the amount of tubulins in the growth cone, as captured by the third term on the right side of Eq. (14). The last two terms describe the assembly/disassembly of microtubules, which have been explained above, referring to Eq. (12). We assume that the axon area *A* and the growth cone volume *V*_c_ are constant during the whole process, and an effective length of growth cone, *l*_c_ = *V*_c_/*A*, can be defined. Therefore, the conservation condition in Eq. (14) leads to the following ordinary differential equation in tubulin concentration:

(15) $$l_{\text{c}}\frac{\text{d}c_{\text{c}}}{\text{d}t}=J_{\text{c}}-r_{\text{g}}c_{\text{c}}\left(c_{\text{c}}-c_{\infty }\right)-gl_{\text{c}}c_{\text{c}}-\tilde{r_{\text{g}}}l_{\text{c}}\left(c_{\text{c}}-c_{\infty }\right).$$

In general, the flux term *J*_*c*_ should be related to the tubulin concentration *c*_c_ and the landscape of potential energy *U*(*x*) through

(16) $$J_{\text{c}}=-D\left(\frac{\partial c_{\text{c}}}{\partial x}+\frac{c_{\text{c}}}{k_{\text{B}}T}\frac{\partial U\left(x\right)}{\partial x}\right),$$

where *D* is the diffusion coefficient. We take an approximation $\frac{\partial c_{\text{c}}}{\partial x}=\frac{c_{\text{c}}-c_{\text{s}}}{l}$ for the concentration gradient as we only care about the influx of tubulin at the growth cone rather than the distribution of concentration along the axon. The gradient of potential energy is assumed to be a constant, which means that tubulins are transported to the growth cone at a constant rate. Furthermore, previous studies have revealed that the transporting rate of tubulins is proportional to the force applied to the axon (O’Toole et al., 2008a, b). Thus, an equivalent description of flux is

(17) $$J_{\text{c}}=v_{0}\bar{F}c_{\text{c}}-D\frac{c_{\text{c}}-c_{\text{s}}}{l},$$

with *v*_0_ being the intrinsic transporting rate when the neuron is under no stretch and $\bar{F}=f\rho_{\text{f}}/f_{0}\rho_{\text{f}0}$ being the normalized force of stress fiber, referring to Eq. (7). Here ρ_f0_ stands for the density of the stress fiber when the neuron is under no stretch.

Substituting the flux term in the ordinary differential equation of concentration [Eq. (15)], we obtain two coupled governing equations for axon elongation with two unknowns, *l* and *c*_c_:

(18) $$\left\{ \begin{array}{c} \frac{\text{d}l}{\text{d}t}=r_{\text{g}}\left(c_{\text{c}}-c_{\infty }\right)\\ l_{\text{c}}\frac{\text{d}c_{\text{c}}}{\text{d}t}=v_{0}\frac{f\rho_{\text{f}}}{f_{0}\rho_{\text{f}0}}c_{c}-D\frac{c_{\text{c}}-c_{\text{s}}}{l}-r_{\text{g}}c_{\text{c}}\left(c_{\text{c}}-c_{\infty }\right)\\ -gl_{\text{c}}c_{\text{c}}-\tilde{r_{\text{g}}}l_{\text{c}}\left(c_{\text{c}}-c_{\infty }\right) \end{array}\right..$$

## Model Parameters

In the preceding sections, we have proposed a theoretical framework that unifies the kinetics of focal adhesion and stress fiber, dynamics of cell reorientation, and elongation of axon/microtubules. We proceed by normalizing the physical variables/parameters in the previous part to obtain dimensionless governing equations that are responsible for cell reorientation and neurite outgrowth. With a normalization scheme

(19) $$\begin{array}{c} \xi =\frac{\rho_{\text{b}}}{\rho_{0}},\eta =\frac{\rho_{\text{f}}}{\rho_{0}},\tau_{1}=k_{\text{on}}t,m=\frac{k_{\text{off}\_0}}{k_{\text{on}}},c=\frac{k_{\text{on}}^{\text{f}}}{k_{\text{on}}},\\ d=\frac{k_{\text{off}}^{\text{f}}}{k_{\text{on}}},v=\frac{\omega }{k_{\text{on}}}, \end{array}$$

Eqs (8) and (9) can be written as

(20) $$\frac{\text{d}\xi }{\text{d}\tau_{1}}=1-\xi -\xi \cdot m\text{exp}\left(-a\frac{\eta }{\xi }+b{\left(\frac{\eta }{\xi }\right)}^{2}\right),$$

(21) $$\frac{\text{d}\eta }{\text{d}\tau_{1}}=c\xi -d\eta .$$

Here $b=\frac{f^{2}}{2k_{\text{B}}T\cdot K}$, with $f=f_{0}+\frac{k_{\text{f}}\varepsilon }{2}(\left(1-\frac{\alpha }{1+\alpha^{2}}\right)e^{-\alpha \left(v\tau_{1}+\varphi \right)}$$+\frac{\alpha }{1+\alpha^{2}}\text{cos}\left(v\tau_{1}+\varphi \right)$$+\frac{1}{1+\alpha^{2}}\text{sin}\left(v\tau_{1}+\varphi \right))$ being the contraction force of stress fiber. The dimensionless parameters and their physical meanings are summarized in Table 2, and these parameters are explored within reasonable ranges to match the experimental observation. For example, the typical value for the on-rate of receptor–ligand bonds is 1–100 s^–1^ (Lawrence and Springer, 1991; Rinko et al., 2004), which is chosen as *k*_on_ = 2πs^−1^ and serves as a reference rate in the model; the off-rate of bonds is usually higher than on-rate by several orders of magnitude, so we choose *m* = *k*_off_0_/*k*_on_ = 150; the single-pair interaction energy between a bond and a reinforcing protein is expected to be a few *k*_B_*T* (Feng et al., 2017), thus *a* = 3.6. The rates of kinetics (association and dissociation) in stress fiber should be lower than those of bonds, as the SF kinetics often involves more complicated processes including actin polymerization and assembly of associated myosin molecules (Hotulainen and Lappalainen, 2006; Endlich et al., 2007). Therefore, the dimensionless parameters *c* and *d* are estimated to be 0.1 and 0.2. The other values for SF parameters, such as *f*_0_ = 1pN, *k*_f_ = 20pN, and η_f_ = 50pN⋅s, are also carefully determined according to previous studies (Balaban et al., 2001; Deguchi et al., 2006; Kumar et al., 2006). We choose the dimensionless parameter $f_{0}^{2}/2Kk_{\text{B}}T=0.9$, which can be interpreted into a spring constant of *K* = 0.135pN/nm.

**TABLE 2:** Physical meanings and adopted values of the involved parameters describing the kinetics of focal adhesion and stress fiber.

Parameter	Meaning	Value
*k*_*on*_	On-rate of receptor–ligand bonds in focal adhesion	2πs^−1^
*m*	Normalized off-rate of receptor–ligand bonds in focal adhesion	150
*c*	Normalized on-rate of filaments in stress fibers	0.2
*d*	Normalized off-rate of filaments in stress fibers	0.1
*a*	Normalized single-pair interaction energy between a bond and a reinforcing protein	3.6
$f_{0}^{2}/2Kk_{\text{B}}T$	Dimensionless parameter representing the substrate compliance	0.9
*f*_0_	Intrinsic force generated by stress fiber	1*pN*
*k*_*f*_	Spring constant of the filament	20*pN*
η_*f*_	Viscous coefficients of the filament	50*pN*⋅s

The normalized governing equations for axon elongation and tubulin concentration are

(22) $$\left\{ \begin{array}{c} \frac{\text{d}L}{\text{d}\tau_{2}}=R_{\text{g}}\left(C_{\text{c}}-1\right)\\ \frac{\text{d}C_{\text{c}}}{\text{d}\tau_{2}}=V_{0}\bar{F}C_{\text{c}}-\bar{D}\frac{C_{\text{c}}-C_{\text{s}}}{L}-R_{\text{g}}C_{\text{c}}\left(C_{\text{c}}-1\right)-C_{\text{c}}-\tilde{R_{\text{g}}}\left(C_{\text{c}}-1\right) \end{array}\right.,$$

with the following normalization scheme:

(23) $$\begin{array}{c} L=\frac{l}{l_{\text{c}}},\tau_{2}=gt,R_{\text{g}}=\frac{r_{\text{g}}c_{\infty }}{l_{\text{c}}g},V_{0}=\frac{v_{0}}{l_{\text{c}}g},C_{\text{c}}=\frac{c_{\text{c}}}{c_{\infty }},\\ C_{\text{s}}=\frac{c_{\text{s}}}{c_{\infty }},\tilde{R_{\text{g}}}=\frac{\tilde{r_{\text{g}}}}{g},\bar{D}=\frac{D}{l_{\text{c}}^{2}g}. \end{array}$$

We also summarize these parameters in Table 3 and collect the reported values for these parameters from the literature. The effective length *l*_c_ should be comparable to the dimension of the growth cone, which is several micrometers (Diehl et al., 2014). The magnitude of tubulin degradation rate in the axon ranges from 10^−7^s^−1^ to 10^−4^s^−1^ (Miller and Samuels, 1997; Caplow and Fee, 2002), and we estimate a larger value of *g* = 0.004s^−1^ because tubulin degradation occurs only in the growth cone in our framework. The disassembly and assembly rates of microtubules are not yet experimentally measured, so we take the values of *R*_*g*_ and $\tilde{R_{g}}$ according to a previous theoretical work (Diehl et al., 2014). The tubulin transport speed for different kinds of animals is 0.5–2 mm/day (Miller and Samuels, 1997; Galbraith and Gallant, 2000), which can be interpreted into the normalized transport velocity of tubulins *V*_0_ ranging from 0.3 to 1.2. For the concentration of tubulins in the soma, we set *C*_*s*_ = 2. The normalized diffusion coefficient of tubulin $\bar{D}=30$ corresponds to *D* = 2×10^−12^m^2^/s, which matches the experimental measurement (Galbraith et al., 1999).

**TABLE 3:** Physical parameters and their values that characterize axon elongation.

Parameter	Meaning	Value
*l*_c_	Effective length of growth cone	4μ*m*
*g*	Degradation rate of free tubulins in growth cone	0.004s^−1^
*R*_*g*_	Normalized disassembly velocity of microtubules	0.16
*V*_0_	Normalized transport velocity of tubulins	0.78
*C*_*s*_	Normalized concentration of tubulins in soma	2
$\tilde{R_{\text{g}}}$	Normalized assembly rate of microtubules	12.5
$\bar{D}$	Normalized diffusion coefficient of free tubulins	*30*

## Results and Discussion

### Stretch Frequency- and Amplitude-Dependent Reorientation of Neuron Cells

**FIGURE 6:**
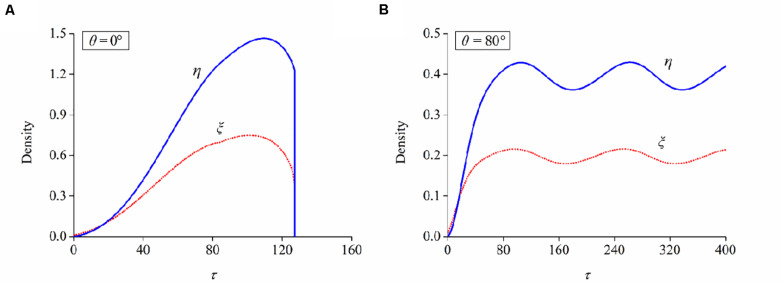
Evolutions of bond and filament densities (ξ and η) when a neuron is aligned at the orientation of **(A)** 0° and **(B)** 80°, respectively. Stretch amplitude: ε = 10%; stretch frequency: ω = 0.25Hz.

We carry out the simulations for neuron reorientation by the following procedure:

(1) First, 100 neuron cells with a uniformly distributed random orientation are generated at time *t* = 0. The initial value of bond density in focal adhesion and filaments in stress fiber for all these cells is selected as ξ = 0.01 and η = 0, respectively.
(2) We monitor the long-term evolution of FA and SF through the time-varying behaviors of ξ and η, when the substrate is under cyclic stretch with specified stretch amplitude and frequency.
(3) Determine which is the case for FA/SF evolution at the present orientation angle: case I—the long-term values of ξ and η cannot maintain a stable level but drop to negligible levels (Figure 6A) and case II—ξ and η increase from the initial value to a plateau level, indicating that these neuron cells can maintain a stable configuration (Figure 6B).
(4) For case II from step (3), the present orientation angle is the final alignment of the corresponding neurons; for case I from step (3), each cell updates its orientation angle by adding an angle hop $\text{d}\theta =N\left(0,1\right)\sqrt{2D_{r}t^{*}}$ before new focal adhesion is nucleated, where *N*(0, 1) is a generated random number following normal distribution with zero mean and unit variance, and the amplitude of this angle hop is set as $\sqrt{2D_{r}t^{*}}=0.5{}^{\circ }$ in the simulation.
(5) For those neuron cells with a new orientation angle, loop from step (2) to step (4) until they find a final orientation at which long-term stability can be achieved in terms of FA and SF (i.e., ξ and η).

**FIGURE 7:**
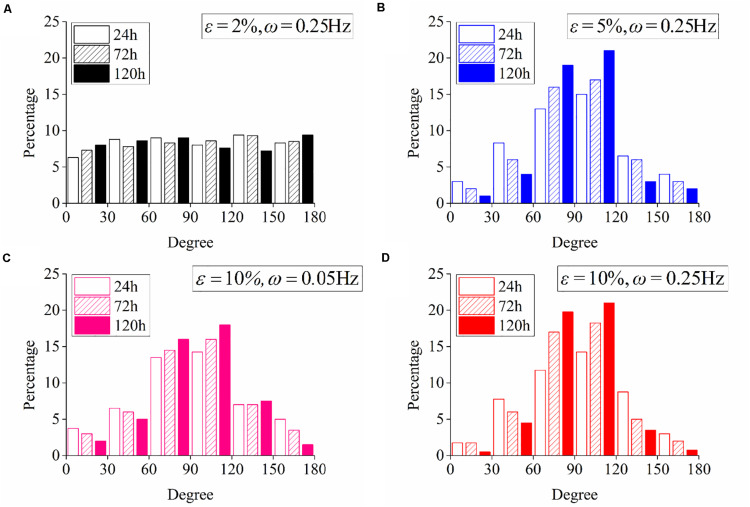
Histograms of model-predicted neuron orientation under cyclic stretch with different combinations of amplitude and frequency. **(A)** ε = 2%, ω = 0.25Hz; **(B)** ε = 5%, ω = 0.25Hz; **(C)** ε = 10%, ω = 0.05Hz; and **(D)** ε = 10%, ω = 0.25Hz.

Following the numerical procedure, we simulate the reorientation process of neuron cells for different cyclic stretch conditions that are used in the experiments (Table 1). Figure 7 plots the histograms of model-predicted cell orientation after 24, 72, and 120 h, when the substrate is subjected to cyclic stretch with different combinations of amplitude and frequency. The simulation results show the same dependence of polarization level on stretch frequency, as observed in experiments (Figure 2). The effect of stretch amplitude on cell reorientation (Figure 3) is also captured here: more neurons align away from the stretch direction (0° in the figure) when stretch amplitude is increased to above 5%. In the simulation, neuron cells do not show a preferable orientation when stretch amplitude is 2% (Figure 7A) because stable FA and SF can be achieved in all orientations when stretch is low enough, without inducing a large force in the stress fiber that hinders the formation of FA and SF. In contrast, when stretch amplitude is 5% or larger, long-term stability in FA and SF is possible only within the angle region close to the direction perpendicular to the stretch (Figures 7B,D). This is because the strain level transmitted to SF/FA depends on the angle between cyclic stretch and neuron orientation, through ε_sf_ = ε_app_cos^2^θ in Eq. (3). When the neuron alignment is close to the stretch direction (i.e., θ is close to zero), the large magnitude of the resultant force on stress fibers tends to hinder the formation of FA and SF. Therefore, FA/SF can only maintain a stable configuration close to the direction perpendicular to the imposed stretch.

**FIGURE 8:**
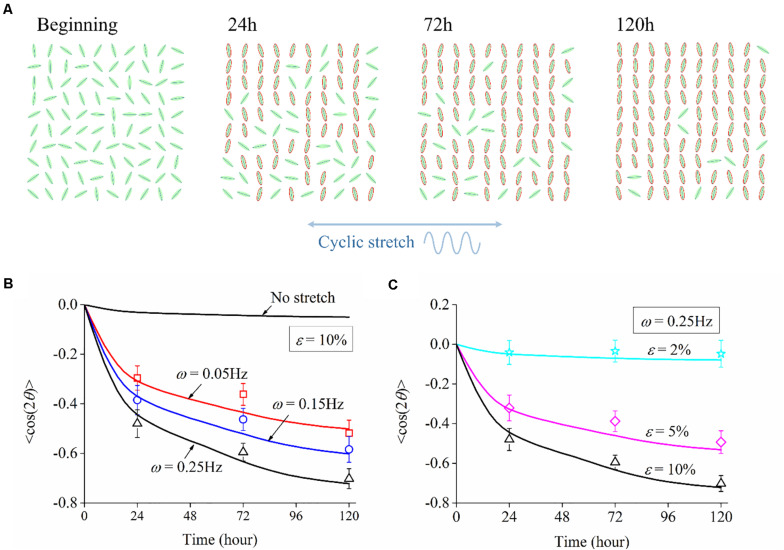
**(A)** Orientation snapshots of 100 individual neuron cells (stretch amplitude: 10%; stretch frequency: 0.25 Hz). Evolution of order parameter, cos2θ(*t*), of the generated neuron cells on substrates subjected to cyclic stretch with different values of **(B)** frequency and **(C)** amplitude. The symbols are experimental data with error bar representing the standard deviation, and the solid curves are simulation results.

We adopt the order parameter ⟨cos2θ(*t*)⟩ that quantifies the instantaneous neuron alignment for these sampled cells. Here θ(*t*) is the orientation angle of each cell at time *t*, which can be obtained from the morphology images in experiments or numerical trajectory in simulation. Taking the simulation with 10% amplitude and 0.25 Hz frequency as an example, neuron cells are randomly orientated at the beginning and rotate themselves to directions nearly perpendicularly to the cyclic stretch as time elapses. After 120 h, 90% of the cells align away from the stretch direction, as highlighted by those neurons with a red boundary in Figure 8A. For the case of no stretch, the order parameter is always near zero; the case of ε = 2% shows a similar result, such that stretch amplitude ε = 2% (or lower) can hardly cause cell reorientation. Figures 8B,C plot the relation of order parameter ⟨cos2θ(*t*)⟩ *versus* time when the substrate is subjected to various frequencies and amplitudes. The order parameter changes from an initial value near zero to a steady-state level: –0.75 for ω = 0.25Hz, –0.55 for ω = 0.15Hz, –0.45 for ω = 0.05Hz in Figure 8B; –0.75 for ε = 10%, –0.5 for ε = 5%, –0.05 for ε = 2% in Figure 8C. The asymptotic behavior of these cases demonstrates that cell reorientation occurs faster when the substrate is subjected to stretch with higher amplitude and frequency.

### Axon Elongation Independent of Cyclic Stretch

**FIGURE 9:**
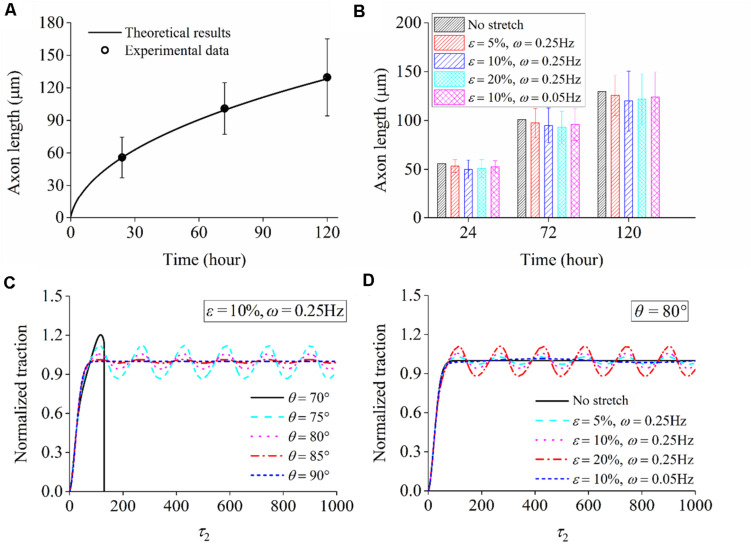
**(A)** Comparison between theoretical predictions and experimental data of axon length when a neuron adheres to the substrate that is not stretched. **(B)** Theoretical results of axon length under cyclic stretch at time 24, 72, and 120 h. Normalized traction force generated in stress fiber: **(C)** when a neuron is aligned along different directions (ε = 10% and ω = 0.25Hz) and **(D)** when the stretch amplitude and frequency adopt different values (orientation angle: θ = 80°).

When neuron cells adhere to the substrate that is not stretched, stable focal adhesion and stress fiber can be formed in all directions, accompanied by a steady–stable value of contraction force in stress fiber. Substituting the parameters in Table 3 and $\bar{F}=1$ into the governing equation [Eq. (22)], we can numerically solve the relation between axon length and elapsed time, which is consistent with the experimental results (open circles in Figure 9A). For the case that the substrate is subjected to cyclic stretch, axon elongation and cell reorientation occur simultaneously. It is conceivable that the disassembly of focal adhesion and stress fiber in cell reorientation does not influence axon elongation much, referring to the towing experiments where neurites unattached to the substrate can grow well with the application of stretch (O’Toole et al., 2008a; O’Toole and Miller, 2011). Figure 9B summarizes axon elongation from our simulation for cases with different stretch amplitudes and frequencies, reproducing the experimental results that axon elongation is independent of cyclic stretch. The contraction force effectively enhances the flux term of tubulins, which is a contributing factor to the evolution and final length of the microtubule/axon through Eq. (18). It is also observed that the level of contraction force in cells along different orientations (Figure 9C) or in different stretching conditions (Figure 9D) is close to that under no stretch because the effective strain transmitted to stress fibers is ε_sf_ = ε_app_cos^2^θ. When θ is close to 90°, as most of the cases in Figures 9C,D (except for θ = 70°), the effective strain on stress fibers is so small and becomes close to the case under no stretch. When θ is reduced to surpass a threshold value between 75° and 70°, the effects of ε_sf_ become significant, and a stable SF/FA configuration cannot be maintained, as reflected by the curve for θ = 70° (Figure 9C) wherein the contraction force drops to zero rapidly.

## Conclusion

By combining *in vitro* cell culture and theoretical modeling of neuron reorientation and axon elongation, this work systematically investigates the effects of cyclic stretch on the morphology of individual neurons. We have developed a feasible device to apply cyclic stretch to neurons through substrate stretching, and it was observed that neurons tend to align away from the stretch direction when stretching amplitude and frequency are sufficiently large. To understand the governing role of stress fiber and receptor–ligand bond cluster in neuron reorientation, we propose a mechanochemical framework that unifies filament assembly/disassembly in stress fiber, bond association/dissociation in focal adhesion, and whole-cell hopping in exploring new orientations. Our model suggests that, when stretching amplitude and frequency are sufficiently large, bond cluster and stress fiber fail to maintain long-term stability for orientations parallel to the direction of cyclic stretch, and neurons tend to rotate to directions that are nearly perpendicular to the stretch direction, consistent with the experimental results.

Our experiments also show that axon elongation in neurons is hardly affected by cyclic stretch. We extend the theoretical framework by including the dynamic evolution of contraction force generated by the stress fiber and the contraction-induced tubulin transportation. It was found that, under cyclic stretch, the average traction level is about the same as the case of no stretch. In other words, axon elongation involves a time scale that is much larger than that in subcellular kinetics, and cyclic stretch with a mean value of zero neither increases nor decreases the effective stress level in neurons. Consequently, the traction-driven transportation of tubulins, serving as a critical step in microtubule assembly in the growth cone and axon elongation, is not affected by cyclic stretch. The present model may serve as a theoretical framework to motivate future studies on neuron orientation, axon outgrowth, and more intriguing processes in neurons under mechanical stimuli.

## Data Availability Statement

The original contributions presented in the study are included in the article/supplementary materials, further inquiries can be directed to the corresponding author/s.

## Author Contributions

JQ and JY conceived and designed the experiments and modeling. JL and JQ derived the theory. XL and JY performed the experiments. JL, XL, JY, and JQ analyzed the data. JL, XL, JY, and JQ wrote the manuscript. All authors contributed to the article and approved the submitted version.

## Conflict of Interest

The authors declare that the research was conducted in the absence of any commercial or financial relationships that could be construed as a potential conflict of interest.
